# Increased susceptibility of spinal muscular atrophy fibroblasts to camptothecin is p53-independent

**DOI:** 10.1186/1471-2121-10-40

**Published:** 2009-05-16

**Authors:** Chia-Yen Wu, Ilsa Gómez-Curet, Vicky L Funanage, Mena Scavina, Wenlan Wang

**Affiliations:** 1 Department of Biological Science, University of Delaware, Newark, DE, USA; 2 Nemours Biomedical Research, Alfred I. duPont Hospital for Children, Wilmington, DE, USA; 3 Department of Pediatrics, Thomas Jefferson University, Philadelphia, PA, USA

## Abstract

**Background:**

Deletion or mutation(s) of the survival motor neuron 1 *(SMN1) *gene causes spinal muscular atrophy (SMA). The SMN protein is known to play a role in RNA metabolism, neurite outgrowth, and cell survival. Yet, it remains unclear how SMN deficiency causes selective motor neuron death and muscle atrophy seen in SMA. Previously, we have shown that skin fibroblasts from SMA patients are more sensitive to the DNA topoisomerase I inhibitor camptothecin, supporting a role for SMN in cell survival. Here, we examine the potential mechanism of camptothecin sensitivity in SMA fibroblasts.

**Results:**

Camptothecin treatment reduced the DNA relaxation activity of DNA topoisomerase I in human fibroblasts. In contrast, kinase activity of DNA topoisomerase I was not affected by camptothecin, because levels of phosphorylated SR proteins were not decreased. Upon camptothecin treatment, levels of p53 were markedly increased. To determine if p53 plays a role in the increased sensitivity of SMA fibroblasts to camptothecin, we analyzed the sensitivity of SMA fibroblasts to another DNA topoisomerase I inhibitor, β-lapachone. This compound is known to induce death via a p53-independent pathway in several cancer cell lines. We found that β-lapachone did not induce p53 activation in human fibroblasts. In addition, SMA and control fibroblasts showed essentially identical sensitivity to this compound. By immunofluorescence staining, SMN and p53 co-localized in gems within the nucleus, and this co-localization was overall reduced in SMA fibroblasts. However, depletion of p53 by siRNA did not lessen the camptothecin sensitivity in SMA fibroblasts.

**Conclusion:**

Even though p53 and SMN are associated, the increased sensitivity of SMA fibroblasts to camptothecin does not occur through a p53-dependent mechanism.

## Background

Spinal muscular atrophy (SMA) is a neuromuscular disease characterized by the loss of spinal motor neurons and muscle atrophy [1]. SMA has an incidence of 1 in 6,000 live births, and is one of the most common genetic causes of infant death [2,3]. Clinically, based on the age of onset and severity of the disease, childhood SMA can be categorized into types I, II, and III [4,5]. Type I patients are diagnosed between the ages of zero to six months and cannot sit unsupported or lift their heads, type II patients are diagnosed between the ages of seven and 18 months and can sit, and type III patients are older than 18 months when diagnosed and can stand alone and walk but may later lose these motor milestones. Although SMA shows a broad spectrum of severity, genetic studies indicate that all clinical phenotypes of SMA are caused by deletion or mutation(s) of the survival motor neuron 1 *(SMN1) *gene [6].

The SMN protein is ubiquitously expressed and localizes in the cytoplasm as well as in the nucleus, where it is usually concentrated in subnuclear structures referred to as "gems" (for Gemini of Cajal bodies [7,8]). The SMN protein plays an essential role in the biogenesis of small nuclear ribonucleoprotein (snRNP) and small nucleolar ribonucleoprotein (snoRNP) complexes [9-11]. SMN appears to perform this function by associating with Gemins 2–8 [12-14]. Recent studies have demonstrated that the associated SMN/Gemin complex directly interacts with specific domains of Sm proteins and uridine-rich snRNAs to ensure stringent control of snRNP assembly [15,16]. In addition to RNP assembly, SMN has been shown to play a role in neurite outgrowth [17,18], through its association with hnRNP R [19,20].

Complete loss of SMN in species ranging from *S. pombe *to mice is lethal, indicating that SMN is critical for survival of multiple cell types [21-23]. More direct evidence to support SMN's role in cell survival comes from studies in cultured cells [24-29]. For example, depletion of the SMN protein in *Drosophila *S2 cells stimulates caspase activity and leads to increased cell death [25]. Importantly, SMN has been shown to play a role in neuronal cell survival. Depletion of SMN in differentiated P19 cells activates caspase activity and increases cell death [27], whereas overexpression of human SMN (hSMN) protects differentiated PC12 cells from cell death induced by neurotrophic factor withdrawal [26].

Previously, we investigated the role of SMN in cell survival using skin fibroblasts derived from SMA patients and age-matched controls [29]. We demonstrated that SMA fibroblasts display an increased sensitivity to camptothecin-induced cell death. Treatment with menadione, an agent causing cell death by generating oxidative stress [30], did not cause differences in survival between SMA and control fibroblasts. In addition, camptothecin treatment resulted in significantly higher caspase-3 activity in SMA fibroblasts when compared with control fibroblasts, and this activity directly correlated with levels of SMN in fibroblasts. Thus, these data support an active role for SMN in cell survival.

Camptothecin is a specific DNA topoisomerase I inhibitor that binds DNA topoisomerase I when the enzyme is complexed with DNA [31]. Consequently, camptothecin stabilizes the enzyme-DNA complex and suppresses the enzymatic activity of this protein. Camptothecin has been shown to induce cell death in human ovarian adenocarcinoma cells via p53-dependent and independent pathways [32]. Interestingly, SMN has been shown to interact with p53, and this interaction is reduced when SMN harbors mutations derived from SMA patients [33]. Because SMN can interact with p53 and camptothecin can induce cell death via p53-dependent and independent mechanisms, this study addresses whether the increased sensitivity of SMA fibroblasts to camptothecin occurs through a p53-dependent mechanism. We found that although SMN directly interacts with p53, the increased sensitivity of SMN-depleted fibroblasts to camptothecin occurs through a p53-independent mechanism.

## Results

### Camptothecin inhibits DNA unwinding but not kinase activity of DNA topoisomerase I in human fibroblasts

**Figure 1:**
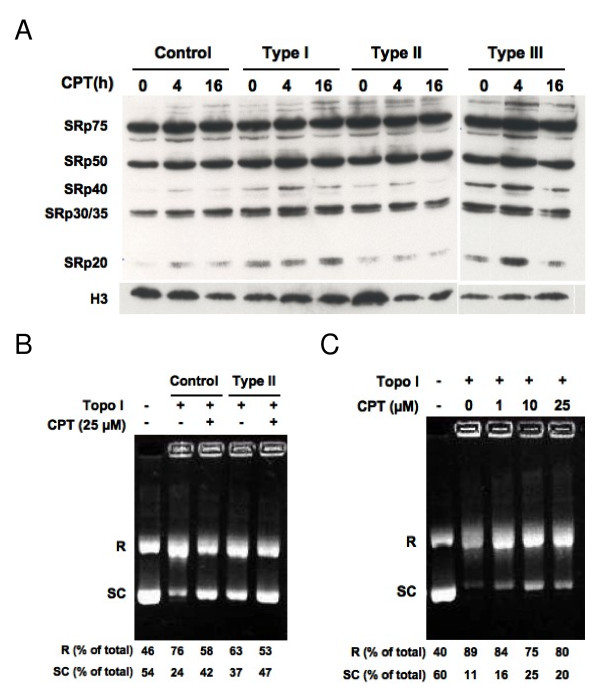
**Camptothecin inhibits DNA relaxation but not kinase activity of DNA topoisomerase I**. *(A) *Control and SMA fibroblasts were treated with 25 μM camptothecin, and levels of phosphorylated SR proteins in the nuclear extracts were analyzed by Western blotting. The same blot was then stripped and reprobed with anti-histone 3 (H3) antibodies as a loading control. *(B) *DNA topoisomerase I was immunoprecipitated from camptothecin treated fibroblasts and subjected to the DNA unwinding assay. Plasmid DNA in the supercoiled form (SC) and in the relaxed form (R) is indicated. *(C) *DNA topoisomerase I immunoprecipitated from untreated control fibroblasts was subjected to the DNA unwinding assay in the presence of camptothecin at the indicated concentrations. All the data shown here are representative of at least two independent experiments. CPT = camptothecin, H3 = histone 3, and Topo I = DNA topoisomerase I.

We previously showed that fibroblasts derived from SMA patients have increased sensitivity to the DNA topoisomerase I inhibitor camptothecin [29]. DNA topoisomerase I has been shown to phosphorylate SR proteins [34] that regulate RNA splicing. Considering SMN's role in RNA splicing, we examined whether camptothecin treatment would block phosphorylation of SR proteins in SMA fibroblasts. Control and SMA fibroblasts were treated with 25 μM camptothecin, and levels of phosphorylated SR proteins in nuclear extracts were analyzed by Western blotting using the mAb 104 antibody that specifically recognizes a phosphorylated epitope at the arginine/serine rich (RS) domain [35]. As shown in Figure 1A, levels of phosphorylated SR proteins were not reduced in camptothecin-treated human fibroblasts. In fact, phosphorylation of some SR proteins was slightly increased, which could be caused by activation of kinases other than DNA topoisomerase I. These data suggest that camptothecin does not inhibit *in vivo *kinase activity of DNA topoisomerase I. Thus, the camptothecin-induced cell death in human fibroblasts must be mediated by suppression of other enzymatic activities of DNA topoisomerase I.

**Figure 2:**
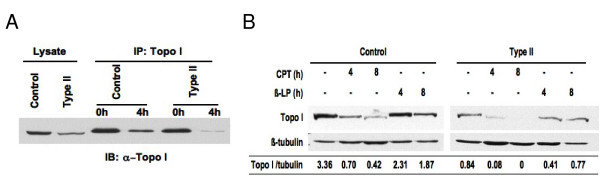
**Camptothecin induces degradation of DNA topoisomerase I in fibroblasts**. *(A) *Presence of DNA toposiomerase I in the immunocomplexes described in 1B was confirmed by Western blotting analyses. *(B) *Fibroblasts described in 1B were treated with 25 μM camptothecin or 25 μM β-lapachone, and DNA topoisomerase I present in the lysates was detected by Western blotting. The same blots were stripped and reprobed with anti-β-tubulin antibodies as a loading control. Relative ratios of DNA topoisomerase I to tubulin levels are indicated. All the data shown here are representative of at least two independent experiments. CPT = camptothecin, Topo I = DNA topoisomerase I, IP = immunoprecipitation, IB = immunoblotting, and β-LP = β-lapachone.

Next, we analyzed the DNA relaxation activity of DNA topoisomerase I from human fibroblasts after camptothecin treatment. Control and SMA fibroblasts were treated with 25 μM camptothecin, and DNA topoisomerase I was immunoprecipitated. DNA relaxation activity of this enzyme was assayed on a supercoiled plasmid DNA [36]. Figure 1B shows that in the absence of DNA topoisomerase I, approximately half of the plasmid DNA was found in the supercoiled form. Upon addition of this enzyme, the majority of plasmid DNA was in the relaxed form, and this DNA relaxation activity was inhibited by camptothecin treatment. When immunoprecipitated DNA topoisomerase I was mixed with camptothecin *in vitro*, DNA relaxation activity of this enzyme was also reduced (Fig. 1C), which is consistent with data obtained from studies with purified DNA topoisomerase I [36,37]. Western blotting analyses indicated that upon camptothecin treatment, levels of DNA topoisomerases I (~100 kD) in the immunoprecipitates and protein lysates were reduced by 80% or more, and SMA fibroblasts had more reduced levels of this enzyme than control fibroblasts (90% [SMA] vs. 80% [control] at 4 h and 100% [SMA] vs. 88% at 8 h) (Figs. 2A and 2B). The reduction in DNA topoisomerase I protein seems specific to camptothecin since another DNA topoisomerase I inhibitor, β-lapachone, did not drastically affect levels of this enzyme (Fig. 2B). β-lapachone directly binds to DNA topoisomerase I and inhibits its enzymatic activity [38]. Thus, *in vivo *inhibition of DNA topoisomerase I by camptothecin likely results from a combination of decreased DNA relaxation activity and reduced levels of this protein.

### Activation of p53 and the sensitivity of SMA fibroblasts to the DNA topoisomerase I inhibitor β-lapachone

**Figure 3:**
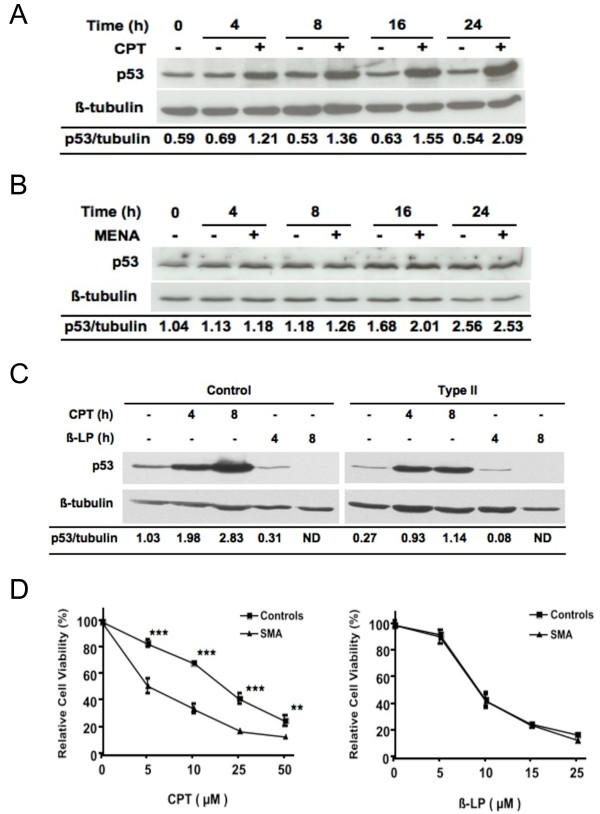
**Activation of p53 and cell death induced by the DNA topoisomerase I inhibitors camptothecin and β-lapachone**. *(A) *A type I SMA fibroblasts as described [29] were treated with 25 μM camptothecin and levels of p53 were analyzed by Western blotting. The same blots were stripped and reprobed with anti-β-tubulin antibodies as a loading control. *(B) *A type II/III SMA fibroblasts as described [29] were treated with 10 μM menadione and levels of p53 were analyzed as described for (A). *(C) *Control and SMA fibroblasts were treated with 25 μM camptothecin or 25 μM β-lapachone and levels of p53 were analyzed as described for (A). Relative ratios of p53 to tubulin levels are indicated. *(D) *Three control and three SMA fibroblasts (one type I, one type II, and one type III) were treated with camptothecin for 72 h or β-lapachone for 24 h at the indicated concentrations, respectively. Cell survival of treated cells was measured by the CellTiter-Blue assay, and the relative cell viability was calculated and presented as percentage of the untreated cells. Each condition was set up as replicates of four and repeated three times. The data presented here are combined mean values ± sem for three control and three SMA fibroblasts. Statistical analyses (unpaired *t *test) indicate that SMA fibroblasts are significantly sensitive to camptothecin at each tested concentration than control fibroblasts (*** *p *< 0.0001 and ** *p *< 0.001). CPT = camptothecin, β-LP = β-lapachone, MENA = menadione, and ND = non-detected.

Camptothecin has been shown to inhibit DNA topoisomerase I activity by stabilizing the enzyme-DNA cleavage complex [31]. As a result, camptothecin treatment results in single- and double-stranded DNA breaks, causes G1 and G2 arrests, and leads to cell death via p53-dependent and independent pathways [32,39]. To elucidate whether the increased sensitivity of SMA fibroblasts to camptothecin is p53-dependent, we assessed p53 induction after camptothecin treatment. As shown in Figure 3A, levels of p53 were markedly increased upon camptothecin treatment, and this induction was seen as early as 4 h and was sustained for 24 h. Treatment with menadione, which also induces death in fibroblasts but does not show differential sensitivity between control and SMA fibroblasts [29], did not elevate p53 levels (Fig. 3B). Unlike camptothecin, menadione causes cell death by generating oxidative stress [30]. This suggested that p53 could play a role in the increased sensitivity of SMA fibroblasts to camptothecin. To further address the role of p53 in camptothecin sensitivity, we examined the sensitivity of SMA fibroblasts to another DNA topoisomerase I inhibitor β-lapachone. This compound has been shown to induce cell death via a p53-independent pathway in several cancer cell lines [40]. Figure 3C shows that levels of p53 protein were not elevated after β-lapachone treatment; and p53 levels actually decreased by more than 70% under this condition. Note that under the same treatment conditions, levels of p53 were elevated two- to four-fold after camptothecin treatment. The SMA fibroblasts showed no increased sensitivity to β-lapachone when compared with control fibroblasts using the percentage of cell death as the readout (Fig. 3D). These data imply that p53 plays a role in the increased sensitivity of SMA fibroblasts to camptothecin.

### Association of p53 and SMN in SMA fibroblasts

**Figure 4:**
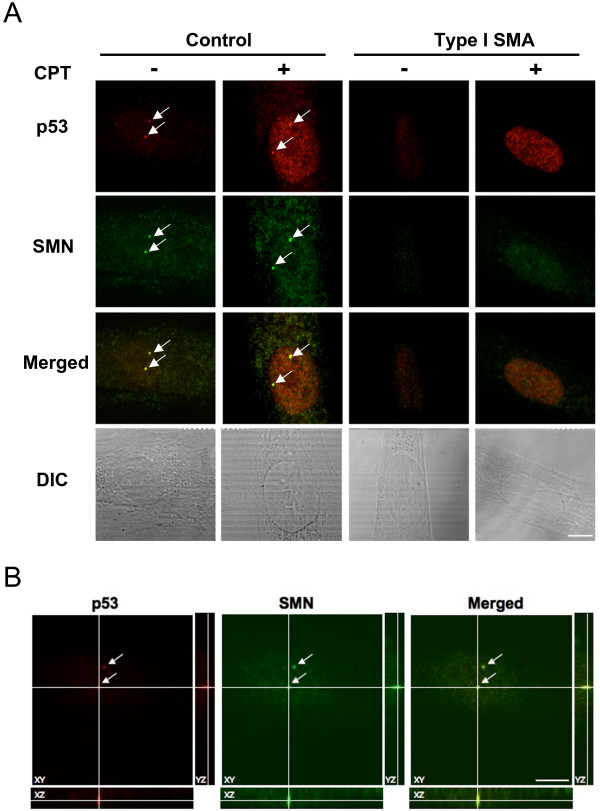
**Association of SMN and p53 in fibroblasts**. *(A) *Control and SMA fibroblasts were left untreated or treated with 25 μM camptothecin for 8 h. Camptothecin-treated cells were fixed and stained with anti-p53 (mouse) and anti-SMN (rabbit) antibodies. Secondary antibodies were either labeled with Alexa 555 (anti-mouse, in red) or FITC (anti-rabbit, in green). Images of p53 and SMN were taken by confocal microscope (63×). Phase contrast images (DIC) for the immunostained cells are included. Scale bar is 10 μm. Gems are indicated by arrows. *(B) *Representative orthogonal views from z-stacking images of untreated control fibroblasts in (A) are shown here. The arrows indicate co-localization between SMN and p53. SMN = survival motor neuron, CPT = camptothecin, and DIC = differential interference contrast.

Since an interaction of p53 with SMN has been previously reported [33], we investigated whether p53 is associated with SMN in fibroblasts. Immunoprecipitation analyses of the endogenous p53 and SMN proteins did not show an association (data not shown), indicating that SMN and p53 interaction in fibroblasts is likely transient and not stable. We next determined if a fraction of endogenous p53 and SMN was associated by using confocal microscopy to test for co-localization. Co-localization of these two proteins was confirmed by visualizing orthogonal projections of stacked images. Control and SMA fibroblasts were left untreated or treated with camptothecin, and p53 and SMN proteins were detected by double-labeled immunofluorescence staining. As shown in Figure 4A, both p53 and SMN were localized in the cytoplasm and the nucleus. Upon camptothecin treatment, p53 staining in the nucleus was dramatically enhanced, which is consistent with the p53 induction measured by Western blotting analyses (see Figs. 3A and 3C). Nuclear immunofluorescence staining showed that SMN was more concentrated in gems, and that SMA fibroblasts had reduced numbers of gems in the nucleus both in the absence and presence of camptothecin treatment (Fig. 4A and Table 1). Gem size in SMA fibroblasts was also smaller than that in control fibroblasts (Table 1), likely as a result of reduced levels of SMN expression in these cells. In addition, SMN and p53 were seen to co-localize in gems in the absence and presence of camptothecin (Figs. 4A and 4B). Overall, co-localization of SMN with p53 was reduced in SMA fibroblasts (Table 1). For example, in the absence of camptothecin treatment, control fibroblasts had 99% gems with SMN/p53 co-localization, whereas SMA fibroblasts had 75%–83%. Upon camptothecin treatment, both number of gems and percentage of gems with co-localized SMN/p53 were reduced in control, type II, and type III SMA fibroblasts. Interestingly, type I SMA fibroblasts had the fewest number of gems, but SMN and p53 co-localized in almost all gems (only one of all counted gems did not contain co-localized SMN and p53).

**Table 1:** Co-localization of SMN with p53 in fibroblasts

	Type I	Type II	Type III	Control
**Untreated**				
Gem number/100 cells	6	16	24	114
Average gem size ± SD (nm)	538 ± 37	603 ± 73	649 ± 121	715 ± 153
% of co-localization with p53	83.3	75.0	75.0	99.1
				
**CPT treated**				
Gem number/100 cells	6	11	17	80
Average gem size ± SD (nm)	544 ± 22	542 ± 53	689 ± 154	793 ± 173
% of co-localization with p53	100.0	63.0	64.7	85.0

### Increased sensitivity of SMA fibroblasts to camptothecin is p53-independent

**Figure 5:**
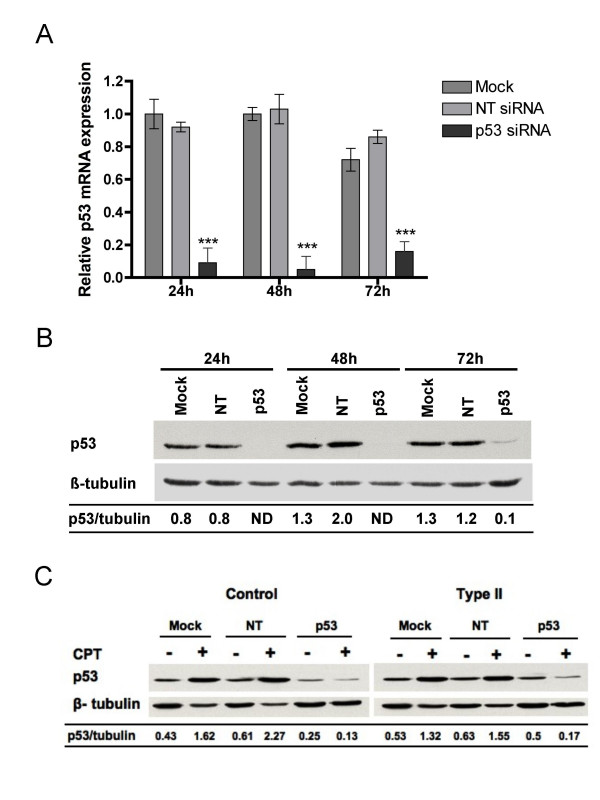
**Depletion of the p53 protein in fibroblasts by RNA interference**. *(A) *Control fibroblasts transfected with p53, non-targeting control, or no siRNA oligonucleotides (mock) were harvested at the indicated times. Levels of p53 mRNA was measured by real-time TaqMan PCR using p53 as the target and gusB as the endogenous control. Statistical analyses (one-way ANOVA) indicate a significant reduction of p53 mRNA by the addition of p53 siRNA at each time point analyzed as compared to mock control (*** *p *< 0.0001). The data shown here are representative of two independent experiments. *(B) *A similar experiment was conducted as described for (A), and levels of the p53 protein were detected as described for 2A. *(C) *Control and SMA fibroblasts were transfected as described for (A). Twenty-four hours after transfection, cells were treated with 25 μM camptothecin for 24 h. Levels of p53 were analyzed as described for (B). NT = non-targeting control, CPT = camptothecin, and ND = non-detected.

**Figure 6:**
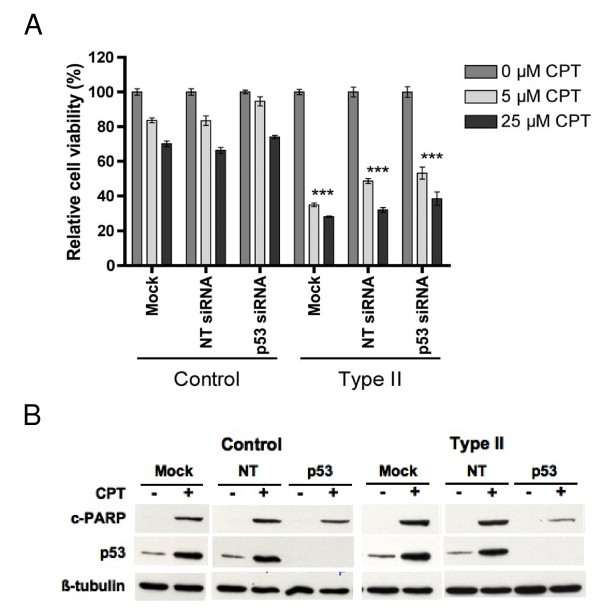
**Depletion of the p53 protein does not lessen the camptothecin susceptibility in SMA fibroblasts**. *(A) *A similar experiment was conducted as described for 5C. Cell survival of treated cells was measured and presented as percentage of the untreated ones. At least two independent experiments were performed and each sample was set up in replicates of four. The mean value ± sem of one representative experiment is presented here. Statistical analyses (unpaired *t *test) indicate that SMA fibroblasts are significantly more sensitive to camptothecin than control fibroblasts (*** *p *< 0.0001). There is no difference in survival among cells transfected with non-targeting control, p53, or no siRNA oligonucleotides for either control or SMA fibroblasts. *(B) *A similar experiment was conducted as described for 5C except cells were treated with 25 μM camptothecin for 16 h. Cell lysates were analyzed by Western blotting using antibodies against cleaved PARP, p53, and tubulin, respectively. NT = non-targeting control, CPT = camptothecin, and cPARP = cleaved poly ADP-ribose polymerase.

Having confirmed the association between SMN and p53 in fibroblasts, we determined if the susceptibility of SMA fibroblasts to camptothecin is mediated by p53. Endogenous p53 protein in fibroblasts was depleted by siRNA, and the sensitivity of SMA fibroblasts to camptothecin was analyzed. Figures 5A and 5B showed a time course for p53 depletion by siRNA in fibroblasts. A reduction of approximately 85–90% in p53 mRNA levels was observed by addition of p53 siRNA nucleotides at each time point analyzed (Fig. 5A). Similarly, levels of the p53 protein were reduced by more than 90% in p53 siRNA transfected cells (Fig. 5B). Levels of p53 in non-targeting control and mock transfected cells were indistinguishable, indicating that p53 depletion by siRNA is specific. Moreover, upon camptothecin treatment, levels of p53 were markedly elevated in fibroblasts, and the increase in p53 expression upon camptothecin treatment was completely eliminated by p53 siRNA (Fig. 5C). Cell survival analyses indicated that SMA fibroblasts were more sensitive to camptothecin than control fibroblasts (~70% survival in control vs. ~35% in SMA fibroblasts after 25 μM camptothecin treatment) (Fig. 6A). Surprisingly, depletion of p53 by siRNA did not rescue either control or SMA fibroblasts from camptothecin-induced cell death. Figure 6A showed that cell death induced by camptothecin was not significantly reduced by p53 depletion. Our previous study showed that SMA fibroblasts have significantly higher caspase-3 activity upon camptothecin treatment than control fibroblasts [29], thus we analyzed induction of camptothecin activated caspase-3 activity in p53 depleted fibroblasts after camptothecin treatment. Figure 6B showed that p53 depletion indeed decreased camptothecin-induced PARP cleavage, an *in vivo *caspase-3 substrate, in both control and SMA fibroblasts. This is consistent with caspase-3 being downstream of p53 [41]. Given that p53 depletion reduced caspase-3 activity but this was not enough to rescue fibroblasts from camptothecin-induced cell death, non-caspase-3 pathways could also be involved in camptothecin-induced death in SMA fibroblasts. When these findings are taken together, we conclude that camptothecin-induced cell death in human fibroblasts is not p53-dependent, and p53 does not play a direct role in the increased sensitivity of SMA fibroblasts to camptothecin.

## Discussion

The critical feature in the pathogenesis of SMA is that reduced levels of the SMN protein result in selective loss of motor neurons accompanied by muscle wasting. SMN has been shown to play a role in the assembly of RNP complexes [9-11], transcription regulation [42], neurite outgrowth [17-19], and cell survival [24-29]. However, how SMN deficiency causes death of motor neurons and muscle paralysis with little effect on other cells and tissues remains unclear. Previously, we demonstrated that SMA fibroblasts are more sensitive to camptothecin-induced cell death when compared with control fibroblasts [29], supporting a role for SMN in cell survival. In this study, we further explored the potential mechanism(s) underlying the susceptibility of SMA fibroblasts to camptothecin.

Camptothecin is a specific inhibitor of DNA topoisomerase I. The best-characterized enzymatic activity of DNA topoisomerase I is the relaxation of supercoiled DNA during DNA replication and RNA synthesis [31]. Camptothecin suppresses the DNA relaxation activity of DNA topoisomerase I by stabilizing the topoisomerase I-DNA cleavage complex [31,43]. As a result, camptothecin treatment introduces single- and double-stranded DNA breaks, leading to chromosome fragmentation and, eventually, cell death [39,43]. The action of camptothecin on the DNA relaxation activity of DNA topoisomerase I has been extensively characterized *in vitro*. Here, we used immunoprecipitated DNA topoisomerase I to analyze *in vivo *DNA relaxation activity in camptothecin-treated human fibroblasts. We found that DNA topoisomerase I from camptothecin-treated fibroblasts had decreased DNA relaxation activity (Fig. 1B), which may be due to reduced levels of this protein and/or reduced enzymatic activity. A reduction in the levels of DNA topoisomerase I in human fibroblasts after camptothecin treatment could be mediated by proteasome-dependent degradation as previously reported for some cancer cell lines [44]. Interestingly, the reduced levels of DNA topoisomerase I after camptothecin treatment were more pronounced in SMA fibroblasts (Figs. 2A and 2B). In addition, in the absence of camptothecin, overall levels of DNA topoisomerase I were lower in SMA fibroblasts than control fibroblasts (Figs. 2A and 2B). Thus, the lower levels of DNA topoisomerase I in SMA fibroblasts could account for their increased susceptibility to camptothecin. Further experiments are needed to determine if the increased sensitivity of SMA fibroblasts to camptothecin is due to lower levels of DNA topoisomerase I in these cells. In addition to its DNA relaxation activity, DNA topoisomerase I exhibits a kinase activity that phosphorylates SR proteins [34]. Since camptothecin treatment of SMA fibroblasts did not result in reduced levels of phosphorylated SR proteins, camptothecin probably did not inhibit kinase activity of DNA topoisomerase I *in vivo*. Thus, inhibition of the DNA relaxation activity of DNA topoisomerase I by camptothecin likely accounts for its cytotoxicity in human fibroblasts.

In human ovarian adenocarcinoma cells, camptothecin has been shown to induce cell death via p53-dependent and independent pathways [32]. The present study showed that levels of p53 were markedly increased in SMA fibroblasts upon camptothecin treatment (Figs. 3A, C, and 4A). Our data also confirmed previous findings [33] in which a fraction of p53 co-localized with SMN in gems, and this co-localization was overall decreased in SMA fibroblasts (Table 1). However, depletion of p53 by siRNA did not lessen the susceptibility of SMA fibroblasts to camptothecin (Fig. 6). Thus, although p53 is activated by camptothecin and p53 and SMN can associate *in vivo*, p53 does not play a direct role in the increased sensitivity of SMN-depleted fibroblasts to camptothecin. This agrees with a previous study performed in SMA transgenic mice [45]. This study indicated that elimination of p53 does not alleviate the disease severity or extend overall lifespan in type I and type III SMA mice. Thus, p53-independent apoptotic pathways may play a role in motor neuron loss when SMN is depleted.

Analyses of the sensitivity of SMA fibroblasts to another DNA topoisomerase I inhibitor, β-lapachone, indicate that SMA and control fibroblasts showed similar sensitivity to this compound (Fig. 3D). β-lapachone is known to induce cell death in several cancer cell lines through a p53-independent pathway [40]. Our data showed that this compound did not induce p53 in fibroblasts (Fig. 3C), suggesting that p53 is not involved in β-lapachone-induced cell death in fibroblasts. Unlike camptothecin, β-lapachone directly binds to DNA topoisomerase I and inhibits its enzymatic activity [38]. Thus, β-lapachone treatment usually does not cause DNA damage [38]. We noticed that β-lapachone did not induce a drastic reduction in the levels of DNA topoisomerase I (Fig. 2B). Since a reduction in DNA topoisomerase I protein by camptothecin seems to be triggered by DNA damage [44], it is possible that β-lapachone did not cause DNA damage in human fibroblasts, so levels of DNA topoisomerase I protein remained unaltered after β-lapachone treatment. Thus, the increased sensitivity of SMA fibroblasts to camptothecin but not to β-lapachone suggests that cell death pathways activated by DNA damage may be responsible for the susceptibility of SMA fibroblasts to camptothecin. SMN may protect fibroblasts from camptothecin-induced cell death through this pathway. This hypothesis is further supported by our observation that in addition to camptothecin, SMA fibroblasts show an increased sensitivity to other DNA damaging reagents (C. Wu, unpublished data). It will be interesting to find out whether other apoptotic molecules in this pathway such as Bax play a role in the vulnerability of SMA fibroblasts to camptothecin, since abolishing this apoptotic protein clearly protects SMA mice from motor neuron loss [46].

## Conclusion

Our results confirm that p53 is activated by camptothecin in human fibroblasts. In addition, p53 co-localizes with SMN in gems, and this co-localization is overall reduced in SMA fibroblasts. However, p53 does not directly affect camptothecin sensitivity when SMN is depleted.

## Methods

### Cell culture and transfection

Skin biopsies from SMA patients and controls were obtained as part of a study approved by the Institutional Review Board of the Alfred I. duPont Hospital for Children. Human fibroblast cell lines were established from these biopsies and maintained according to standard protocols [29]. In brief, fibroblasts were maintained in DMEM supplemented with 20% fetal bovine serum and antibiotics. Passage numbers for control and SMA fibroblasts were matched as closely as possible for all experimental procedures and always kept ≤ #25. These fibroblasts were used in our previous studies [29,47]. Unless specifically denoted, SMA fibroblasts used in this study are not the cell lines used in our initial published study [29]. The number of *SMN1* and *SMN2* gene copies for control and SMA fibroblasts were determined by quantitative real-time PCR as described [47]. Control fibroblasts carry two copies of *SMN1* and two copies of *SMN2*. All SMA fibroblasts have zero copies of *SMN1*. For the *SMN2* gene, most type I fibroblasts contain two copies, type II mainly carry three copies, and type III carry three or more copies. For RNA interference (RNAi) analyses, 1 × 10^6 ^fibroblasts were electroporated with 100 nM of small interference RNA (siRNA) oligonucleotides in nucleofector solution optimized for primary fibroblasts following manufacturer's instruction (Amaxa, Gaithersburg, MD).

siRNA oligonucleotides (SMARTpool kits) for human p53 (p53 SMART pool, L-003329) and non-targeting control (siGenome non-targeting siRNA, D-001206-14) were purchased from Dharmacon (Chicago, IL).

### Analysis of p53 transcript levels by real-time PCR

For p53 RNAi validation, control fibroblasts were transfected with no siRNA (mock), non-targeting control, or p53 siRNA oligonucleotides. Cells were harvested at 24, 48, and 72 h after transfection. Total RNA was isolated using the RNeasy kit with on-column DNase treatment (Qiagen, Los Angeles, CA). First-strand cDNA synthesis was carried out with the Omniscript kit (Qiagen). The real-time PCR was performed in a total volume of 15 μl, containing 10 ng of cDNA, 1× TaqMan Universal PCR master mix (Applied Biosystems, Atlanta, GA), and 1× p53 gene expression assay (Hs01034253) from Applied Biosystems. The real-time PCR was performed on a 7900 HT Sequence Detection System (Applied Biosystems) using a 384-well format, and amplification was achieved using the standard amplification protocol. To enable normalization of the input target cDNA added to each well, the endogenous control GusB (GusB gene expression assay, 4333767F, Applied Biosystems) was amplified simultaneously in a separate reaction well but under identical thermal cycling conditions. Each reaction was run in triplicate and each sample was run at least twice. Amplification data were analyzed using the Sequence Detection Software SDS 2.2 (Applied Biosystems) and running relative quantification (RQ) studies where p53 was identified as the target and GusB as the endogenous control.

### Western blotting analyses and immunoprecipitation

For p53 RNAi validation at the protein levels, control fibroblasts were transfected with no siRNA (mock), non-targeting control, or p53 siRNA oligonucleotides. Cells were harvested at 24, 48, and 72 h after transfection. Lysates from fibroblasts were prepared, protein concentration was measured by the BCA assay, and Western blotting analyses were performed as previously described [29]. In brief, 50 μg of protein lysates was resolved on 7.5% SDS-PAGE for DNA topoisomerase I detection, 10% SDS-PAGE for phosphorylated SR proteins, histone 3 (H3), and cleaved PARP detection, or 12% SDS-PAGE for p53, SMN, and β-tubulin detection. Blots were probed with antibodies against DNA topoisomerase I (1:50, hybridoma 8G6 supernatant, a kind gift from Dr. Daniel Simmons at the University of Delaware, USA) [37]), phosphorylated SR proteins (mAB 104, 1:1000, a kind gift from Dr. Paula Grabowski at the University of Pittsburgh, USA) [35]), histone 3 (1:1000, Cell Signaling, Danvers, MA), cleaved PARP (1:200, Millipore, Billerica, MA), p53 (1:500, Santa Cruz Biotechnology, Santa Cruz, CA), SMN (1:1000, BD Sciences, San Jose, CA), and β-tubulin (1:500, Santa Cruz). The blots were then incubated with the appropriate secondary HRP-conjugated antibodies, and proteins were detected using enhanced chemiluminescence (AmershamPharmacia). Signals were quantified using Image J (National Institute of Health, Bethesda, MA). The ratios of p53 or DNA topoisomerase I to tubulin levels were calculated.

For immunoprecipitation of human DNA topoisomerase I, fibroblasts were left untreated or treated with 25 μM camptothecin for 4 or 8 h. Cell lysates were prepared, and 750 μg of cell lysates in 1 ml of lysis buffer as described above was incubated with 2.5 μg of purified monoclonal anti-DNA topoisomerase I antibody 8G6 plus protein A/G beads (Santa Cruz) at 4°C overnight. The immunocomplex was extensively washed with lysis buffer and then with DNA relaxation assay buffer and subjected to DNA unwinding assay (see below), or eluted with SDS sample buffer, which preceded Western blotting analyses. Similar results were obtained for both time points, and only results obtained at 4 h are shown in Figure 2A.

### DNA unwinding assays

Fibroblasts were left untreated or treated with 25 μM camptothecin for 4 or 16 h. DNA topoisomerase I was immunoprecipitated and assayed for DNA unwinding activity as described [36]. In brief, immunoprecipitated DNA topoisomerase I was incubated with 1 μg of pBluescript plasmid DNA (Stratagene, La Jolla, CA) in 20 μl of relaxation buffer (10 mM Tris-HCl, pH 7.5, 50 mM KCl, 5 mM MgCl_2_, 0.1 mM EDTA, 0.5 μg/ml BSA, and 0.2 mM DTT) for 30 min at 37°C. The reaction was stopped by adding 6 μl of loading buffer containing 50 mM EDTA, 0.5% SDS, 0.1% bromophenol blue, and 50% (w/v) sucrose. The samples were separated by electrophoresis in 1% agarose gels in TBE buffer (30 mM Tris base, 90 mM boric acid, and 2 mM EDTA, pH 8.0). DNA bands were visualized by ethidium bromide staining. Similar results were obtained for both time points, and only results obtained at 4 h are shown in Figure 1B. To assess *in vitro *the inhibitory effect of camptothecin on enzymatic activity, the immunoprecipitated DNA topoisomerase I from untreated fibroblasts was incubated with plasmid DNA in the presence of camptothecin, and the DNA unwinding activity was assayed as described above.

### Nuclear extract preparations

Control and SMA fibroblasts seeded on 100-mm dishes at a density of 1 × 10^6 ^per dish were left untreated or treated with 25 μM camptothecin. Treated cells were harvested at 0, 4, and 16 h after treatment, and resuspended in hypotonic lysis buffer (10 mM HEPES, pH 7.9 containing 1.5 mM MgCl_2_, 10 mM KCl, 0.2 mM PMSF, and 0.5 mM dithiothreitol). Cells were allowed to swell for 10 min and after that homogenized. The nuclei were collected by centrifugation, and resuspended in lysis buffer as described above. After 20-min incubation on ice, lysates were centrifuged at 13,200 rpm for 15 min at 4°C, and the protein concentration of nuclear extracts was measured by the BCA assay. Fifty micrograms of nuclear extracts was subjected to Western blotting analyses using antibodies against the phosphorylated SR proteins and histone 3.

### Chemical treatments and cell survival assays

For p53 activation analysis, approximately 1 × 10^6 ^fibroblasts were seeded on 100-mm dishes. Eighteen hours after seeding, cells were left untreated or treated with camptothecin (Sigma, St. Louis, MO), β-lapachone (Biomol, Plymouth Meeting, PA), or menadione (Sigma) at the indicated time points (Fig. 3). Activation of p53 was analyzed by Western blotting (see above). For analysis of cell survival, fibroblasts were seeded on 96-well plates at a density of 3 × 10^4 ^cells/well. Eighteen hours after seeding, cells were washed three times with 0.5% BSA in DMEM and exposed to either camptothecin for 72 h or β-lapachone for 24 h at the indicated concentrations (Fig. 3D). Cell viability of treated cells was measured by the CellTiter-Blue assay following manufacturer's recommendations (Promega, Madison, WI). All treatment conditions were set up on three control and three SMA fibroblasts and each condition was assayed in quadruplicate. The relative cell viability was calculated for each condition. The results for three control and SMA fibroblasts were combined and presented as the percentage of the untreated cells. For cell survival analyses in p53-depleted fibroblasts, approximately 6 × 10^4 ^transfected fibroblasts were seeded on 96-well plates. Twenty-four hours later, cells were treated with 5 or 25 μM camptothecin for 24 or 48 h. Cell survival was measured by the CellTiter-Blue assay (Promega). Similar results were obtained for both time points, and only results obtained at 24 h are shown in Figure 6A. For the activation of caspase-3 in p53-depleted fibroblasts, approximately 1 × 10^6 ^transfected cells were seeded on 100-mm dishes. Twenty-four hours later, cells were treated with 25 μM camptothecin for 16 h, and the activation of caspase-3 *in vivo *was analyzed by detection of cleaved PARP by Western blotting as described above.

### Immunofluorescence

Fibroblasts were washed in phosphate-buffered saline (PBS) and fixed with 4% paraformaldehyde in PBS for 10 min at room temperature. After two washes with PBS, cells were permeabilized with 0.2% Triton X100 for 15 min, washed twice with PBS, and blocked with 5% bovine serum albumin in Tris-buffered saline containing 0.05% Tween-20 (TBST) for 1 h. Cells were then incubated overnight at 4°C with polyclonal anti-SMN antibodies (1:100, Santa Cruz), or monoclonal anti-p53 antibody (1:100, Santa Cruz) diluted in blocking buffer. After three TBST washes, the cells were then incubated with Alexa fluor-555 conjugated anti-mouse antibodies (1:600, Invitrogen, Chicago, IL) or FITC-conjugated anti-rabbit antibodies (1:200; Jackson ImmunoResearch Laboratories, West Grove, PA) for 1 h and mounted on glass slides using Vectashield (Vector Laboratories, Southfield, MI). Serial images were taken on a confocal TCS-SP2 laser-scanning microscope with overlapped excitation and emission wavelength removed (Leica Microsystems, Inc., Bannockburn, IL). Co-localization of SMN and p53 was visualized in *x*-, *y*-, and *z*-planes using orthogonal views of stacked images. Number of gems with or without co-localized SMN/p53 per 100 cells was counted in control and each type of SMA fibroblasts. Only gems sizes ≥ 0.5 μm in diameter were included because they can be easily detected on a single orthogonal plane.

## Authors' contributions

CW carried out cell survival, immunofluorescence staining, Western blotting studies, and statistical analyses. She also participated in writing of the manuscript. IG gave valuable suggestions in experimental designing, carried out real-time PCR, and helped with writing of the manuscript. VF and MS recruited control and SMA fibroblasts and participated in the writing of the manuscript. WW carried out nuclear extract preparation and helped with Western blotting analyses. WW also participated in the overall design of the study and the writing of the manuscript. All authors have read and approved the final manuscript.
